# Understanding AI Acceptance in Healthcare and ‘Glue Ear’ Diagnosis: A Survey of Clinicians and Service Users

**DOI:** 10.3390/audiolres16050157

**Published:** 2026-10-10

**Authors:** Alexis Bennett, Fei Zhao, Henry Yeomans, Andrew Hall, Wen Jiang

**Affiliations:** 1 Department of Applied Psychology, Cardiff School of Sport and Health Sciences, Cardiff Metropolitan University, Cardiff CF5 2YB, UK; 2 Centre for Speech and Language Therapy and Hearing Science, Cardiff School of Sport and Health Sciences, Cardiff Metropolitan University, Cardiff CF5 2YB, UK; 3 Public Involvement Consultancy, Cardiff CF5 2YB, UK; 4 ENT Department, University Hospital of Wales, Cardiff & Vale University Health Board, Cardiff CF5 2YB, UK; 5 The First College of Clinical Medicine, Xuzhou Medical University, Xuzhou 221000, China; 6 Department of Otolaryngology, The Affiliated Hospital of Xuzhou Medical University, Xuzhou 221000, China

**Keywords:** artificial intelligence, healthcare, glue ear, otitis media with effusion (OME), acceptance, clinician, patient

## Abstract

**Background:** This pilot survey explored the views of clinicians and service users regarding the use of AI in a specific diagnostic scenario of OME. **Methods:** An online survey was conducted in collaboration with a Patient and Public Involvement and Engagement panel and advertised through research and clinical networks, social media platforms. Participants (N = 95; 21–72 years) included clinicians (59%) and service users (32%; patients and carers) with experience of OME. Quantitative and qualitative data were collected and analysed, comparing the views of the two groups across the two contexts. **Results:** Participants demonstrated a generally positive view of AI in OME diagnostics and in healthcare. Clinicians and service users reported comparable levels of trust and acceptance, although clinicians expressed greater concerns about flexibility and accuracy. Perceived key benefits included time and cost savings, while transparency and trust appeared as pivotal facilitators for acceptance. Frequent concerns centred on operational reliability and ethical considerations, with less emphasis on data confidentiality. **Conclusions:** The findings indicate broad support for AI applications both in OME diagnosis and more widely in healthcare, contingent on addressing concerns about accuracy, human oversight, and ethical use. Recommendations include focused communication strategies emphasising transparency and human oversight in the clinical decision-making processes.

## 1. Introduction

The integration of artificial intelligence (AI) into healthcare services has accelerated in recent years, significantly enhancing various aspects of medical practice. AI technologies are now widely applied across routine healthcare tasks and data management, transforming healthcare operations and workflows [1].

Recent studies have increasingly shown that AI-driven diagnostic tools can effectively classify diseases across a range of clinical scenarios [2], particularly in image-based detection [3] such as breast cancer screening [4]. This advancement in medical imaging holds remarkable promise for enhancing diagnostic accuracy, facilitating early disease detection, and ultimately leading to improved patient outcomes and healthcare efficiency [5,6]. In the meantime, by minimising unnecessary tests, misdiagnosis, and delayed intervention, efficient and accurate AI-powered diagnostic tools can contribute to substantial cost savings in healthcare [7].

OME can be challenging to identify because symptoms may be subtle, fluctuate over time, and occur without the pain or fever typically associated with acute ear infection. Delayed recognition may prolong hearing difficulties and can adversely affect speech and language development, educational participation, social functioning, and quality of life in affected children [8,9,10]. Accurate assessment may also be difficult in primary care, where access to specialist audiological expertise and equipment is limited. AI-assisted analysis of objective WAI measurements could therefore support more consistent initial assessment and facilitate appropriate referral where specialist evaluation is required.

To address the challenges of diagnosing OME, AI-powered diagnostic tools have been developed using Wideband Absorbance Immittance (WAI), a fast, non-invasive method for evaluating middle-ear function. Using retrospectively collected WAI data from ears classified as normal or as having OME on the basis of otoscopy and conventional tympanometry, machine-learning models have achieved classification accuracies of 74% to 82% [11] and, using a two-stage deep-learning approach, of over 90% [12]. However, these AI results reflect model development and internal validation on previously labelled data sets from hospital settings. The diagnostic accuracy of the AI-WAI system when applied prospectively to unselected children in primary care, and against an accepted reference standard, has not yet been established. Prospective evaluation of the AI-WAI system in real-world clinical settings is therefore essential to determine its effectiveness in identifying OME in children in comparison with conventional diagnostic methods.

The AI-WAI system is intended as a diagnostic support tool for use at the first point of contact, including primary-care and community settings where specialist audiological expertise may be limited. WAI is obtained using a probe placed in the ear canal and provides an objective assessment of middle-ear function. The AI model analyses the multidimensional WAI pattern and indicates whether it is consistent with OME or with normal middle-ear function. Depending on the local clinical pathway, the measurement could be performed by an appropriately trained healthcare professional, including an audiologist, clinical scientist, primary-care clinician, nurse, or other trained practitioner. The AI output is intended to support, rather than replace, clinical assessment and should be interpreted alongside the patient’s symptoms, clinical history, otoscopic findings, and other relevant information, with the responsible healthcare professional retaining responsibility for final diagnostic, management, and referral decisions [10].

However, despite strong evidence supporting AI-assisted diagnosis and the readiness of some AI systems for integration into primary healthcare, acceptance among healthcare professionals remains a challenge. A degree of caution or reluctance persists, particularly in the absence of established guidelines for clinical use. In the meantime, recent literature suggests that AI applications in primary care are still underdeveloped, mainly as a result of a lack of awareness and understanding of their benefits and capabilities [13,14,15]. A key factor in the successful implementation of AI-based diagnostic tools is the perception of AI among clinicians and service users. Without sufficient levels of acceptance of the technology, the integration of AI-based diagnostic tools into healthcare is unlikely to succeed [16]. Accordingly, successful implementation of OME diagnosis technology requires consideration of the perceptions of stakeholders and effective information dissemination strategies, guided by empirical evidence.

Acceptance of AI in healthcare remains variable and highly context-dependent. Clinicians do not view AI uniformly, and its perceived value depends on how it affects diagnostic accuracy, workflow efficiency, and decision-making in practice. As reported by Lambert et al. [16], concerns about integration into clinical workflows and potential loss of professional autonomy are common, while appropriate training can improve acceptance. Patient perspectives are often less well represented. Moy et al. [17] noted that current AI applications do not always align with patients’ needs and expectations. Public awareness also remains superficial, as many individuals are familiar with AI in general but lack understanding of its specific clinical applications [18]. This trust is not based on a single factor, but on whether AI systems are reliable, transparent, usable, and ethically sound [19]. These elements determine whether AI is seen as a useful support or a potential risk in decision-making.

Recent work further illustrates the rapid development of AI-based clinical decision support and otologic applications, including updated evidence on clinician attitudes toward AI-based decision-support systems [20], machine-learning applications in otology and neurotology [21], and AI models for OME detection [22,23]. These developments reinforce the need to evaluate not only technical performance but also stakeholder perceptions and implementation requirements.

Furthermore, acceptance also depends on whether the technology demonstrates clear clinical benefit and fits the specific context in which it is used. It is shaped by perceived usefulness, trust, and contextual relevance, with variation across user groups and clinical applications [24]. Consistent with this, recent evidence shows that both clinicians and patients are more willing to engage with AI when its value is evident, its limitations are understood, and its role is clearly defined [25,26]. Experience across settings suggests that AI adoption is shaped by a balance between perceived benefit and perceived risk. The same system may be accepted or resisted depending on how well it aligns with clinical needs, user expectations, and the realities of everyday practice.

The aim of the current pilot survey was to explore the perceptions of clinicians and service users regarding the use of AI specifically in the context of the novel AI-based diagnostic system for OME, and to compare these to their views regarding the use of AI in healthcare in general, given prior evidence suggesting that acceptance varies across clinical contexts. In this study, acceptance refers to stated or prospective acceptance, that is, participants’ expressed approval of and willingness to accept the AI-based diagnostic support described in a hypothetical scenario, rather than demonstrated adoption following actual use [27]. The observed aspects involved familiarity, level of acceptance, perceived benefits, concerns, trust, and factors facilitating the acceptance of AI. By generating valuable insights into perceptions of AI applications in OME diagnosis and in healthcare, these results will contribute towards effective implementation and communication strategies for AI both in audiology and more generally in healthcare.

## 2. Methods

The reporting of this survey was guided by the Consensus-Based Checklist for Reporting of Survey Studies (CROSS) [28]. This was a cross-sectional online survey conducted in Qualtrics on 17 June 2024–14 October 2024.

### 2.1. Participants and Recruitment

Eligible participants were adults aged 18 years or older with some awareness of AI.

Clinicians were eligible if they had experience within NHS hospital, community, audiology, paediatric, ENT, or primary-care settings, were familiar with UK primary care, and had experience managing patients with OME. In this study, the term ‘clinicians’ predominantly referred to audiologists and clinical scientists with NHS experience, together with a smaller number of medical and allied healthcare professionals.

Service users were eligible if they were parents or carers of children who had experienced OME or had experienced OME themselves. No additional formal exclusion criteria were applied beyond these eligibility requirements, and eligibility was based on self-report. There were no restrictions on participants’ current location or country of residence.

Participants were recruited via voluntary response and snowball sampling, using emails, letters, electronic posters, and social media posts. Recruitment channels included a university-wide online platform for staff and students, the networks of the research team and the PPIE panel, social media platforms, and relevant audiology- and healthcare-related associations’ websites and newsletters in the UK.

While the exact size of the target population was unknown, it was expected to be relatively small and hard to reach within the pilot study’s limited, funding-constrained data collection period, which also coincided with a common holiday season. Given the exploratory nature of the study and these practical constraints, a pragmatic sample-size target was adopted, using a 90% confidence level, 0.5 standard deviation, and 10% margin of error, yielding a minimum target of 68 completed responses. This target was intended to support preliminary descriptive and exploratory analyses rather than definitive population-level inference or adequately powered subgroup comparisons. Open-ended questions were also included to provide complementary qualitative insights to support interpretation of the quantitative findings.

### 2.2. Summary of Questionnaire Development

The questionnaire was collaboratively developed by the research team and the Patient and Public Involvement and Engagement (PPIE) panel, informed by established guidelines [29,30] and prior research on AI acceptance in healthcare, while incorporating stakeholder-informed perspectives. It underwent two rounds of pilot testing with a group of 11 individuals, including researchers in audiology, health sciences, psychology, and service users with clinical backgrounds. Feedback was used to refine the questionnaire’s structure, clarity, and content. Detailed wording of all survey items and the case scenario of the use of AI to support initial OME diagnosis is provided in Supplementary Materials S1 [31,32,33,34,35]. Descriptions and rationale for individual items are summarised in Table 1.

Before answering the OME-specific questions, all participants were presented with the same case scenario (Supplementary Materials S1). The scenario described a community paediatrician assessing a 5-year-old child with frequent ear problems and hearing difficulties. Assessment of the child’s symptoms and medical history was followed by a simple, quick, and non-invasive hearing test. Participants were told that the test results would be interpreted by an AI-based diagnostic system, which, in the illustrative case, suggested an 85% likelihood of OME (‘Glue Ear’). The accompanying explanation described the system as combining a simple hearing-test device with user-friendly graphical software that automatically generated a diagnostic suggestion from measurements obtained using a probe placed in the ear canal. The profession of the person physically performing the measurement and responsibility for the final diagnosis, management, and referral decisions were not explicitly specified in the scenario.

The final survey questionnaire is summarised below, comprising three sections:

- **Section 1:** Participant role in relation to OME (e.g., clinician, patient, carer), consent form, withdrawal code, and clinician-specific profession details.
- **Section 2:** Questions on views about AI in healthcare, including a case scenario, familiarity with AI, and a series of evaluation and factor-based items (see Table 1 and Supplementary Materials S1 [31,32,33,34,35]).
- **Section 3:** Demographic information (e.g., age, gender, ethnicity, country), repeated withdrawal code, and open comments.

The survey used 4- to 7-point Likert scales and multiple choice questions, including “Prefer not to state” and “Other” with free text. To reduce bias, Likert scale options were ordered from unfavourable to favourable. Only one question (Q2) was mandatory to ensure meaningful engagement, while other items were optional. Matrix questions were avoided to improve accessibility.

### 2.3. Ethical Considerations

The study was approved by the Ethics Committee of Cardiff School of Sport & Health Science (Sta-9413). The survey was entirely anonymous as no personal identifiers were collected and any potential identifiable information in the free-text responses were anonymised. Participation was voluntary, no incentives were offered, and participants were provided with an anonymous withdrawal code, presented after consent and again at the end of the survey, which could be used to withdraw their responses within a 14-day period.

### 2.4. Analysis

Descriptive statistics were used to summarise participant responses. Likert-scale items were coded numerically to reflect levels of familiarity and evaluation, with higher values indicating stronger familiarity or more favourable views. Likert-type responses were treated as ordinal data. Medians were used as the primary measure of central tendency, while means and standard deviations were additionally reported as supplementary descriptive summaries to facilitate comparison with previous survey-based studies. Means and standard deviations were not used as the basis for parametric inference. Accordingly, all inferential analyses involving ordinal responses were conducted using nonparametric methods, including Wilcoxon rank-sum tests for group comparisons and Spearman’s rank correlations for associations. Furthermore, binary-coded factor selection questions were analysed using frequency distributions to assess the relative importance of each factor. Differences in selection frequencies across related items were assessed using the Friedman test, with post hoc sign tests where applicable. In subcases involving small samples, binomial tests were used. Bonferroni correction was applied to control for Type I error where appropriate.

Free-text responses were analysed using thematic analysis following Braun and Clarke’s [36] framework. Responses were coded inductively and grouped into key themes, with illustrative quotes extracted. Specific statistical analyses are presented in the relevant sections of the Results.

## 3. Results

### 3.1. Respondent Characteristics and Associated Viewpoint Differences

**Figure 1:**
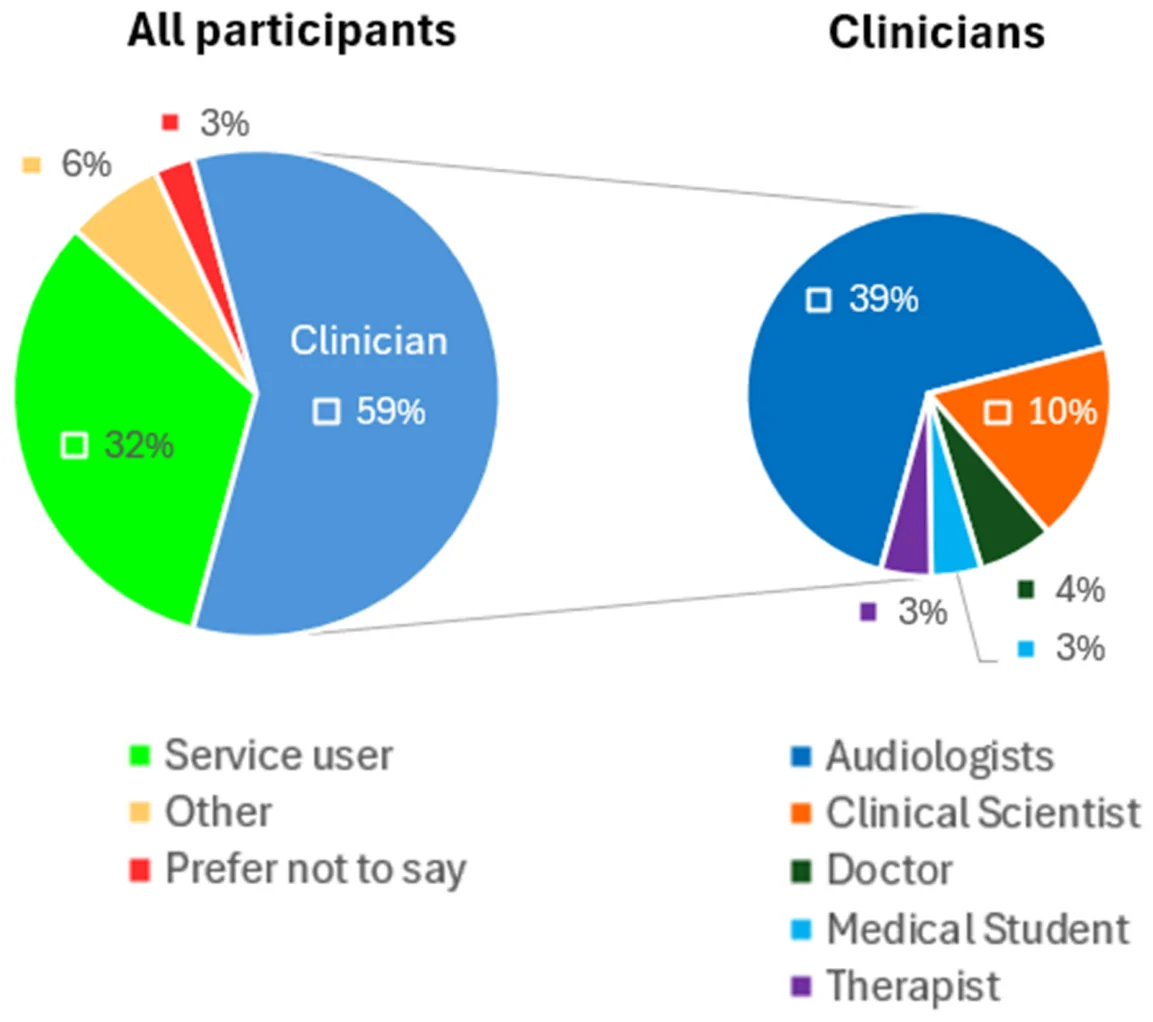
Characteristics and distribution of the survey participants.

Of the 166 people who accessed the survey, 95 (57.2%) gave consent and proceeded. Of these, 78 (82.1%) completed all the questions, while the remaining 17 completed an average of 45%. For those who completed the full survey, the age range was 21 to 72 years (average 38) and gender 70.5% female, 26.9% male, 2.6% nonbinary/other/not stating. Ethnicity was mainly White (62.8%) or Asian (29.5%), with 7.7% mixed/other ethnicity/not willing to state. Country of residence was divided between Wales (44.9%) and England (43.6%), with 11.5% stating Scotland or other. The majority of participants were clinicians (59.0%; N = 46), followed by service users (32.1%; N = 25) and others or unstated (9.0%) (Figure 1). Most clinicians were audiologists (66.7%; N = 30; Figure 1). Among the 25 service users, 15 (60%) were carers and 10 (40%) were patients. These two subgroups were combined for the main analyses because of their small sample sizes.

**Figure 2:**
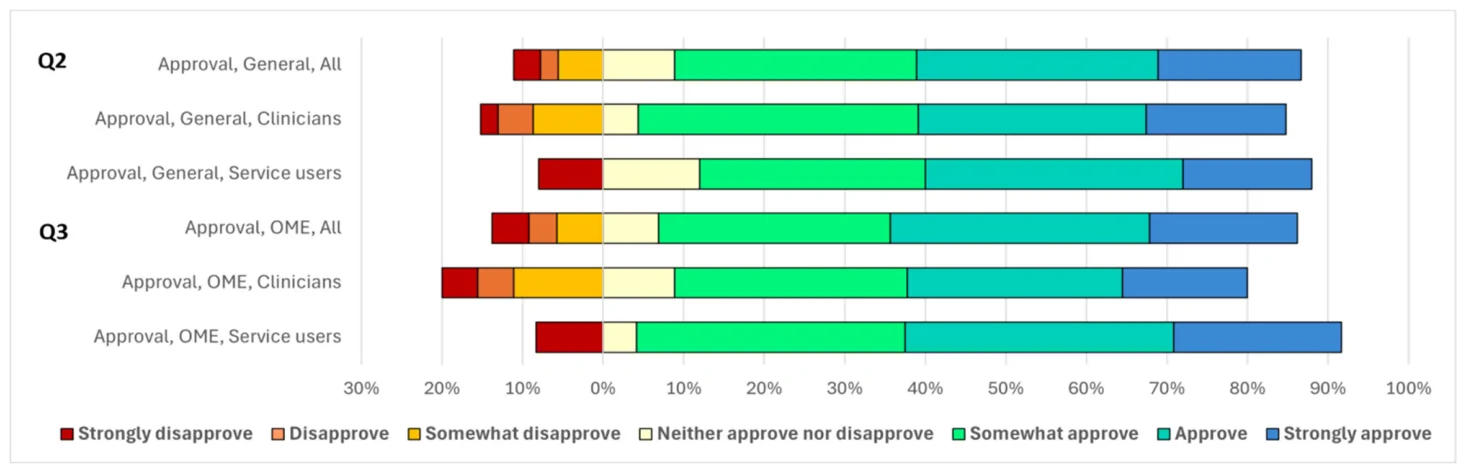
Approval of AI in healthcare, in general (Q2), and in the diagnosis of OME (Q3): All participants, Clinicians, and Service users.

**Figure 3:**
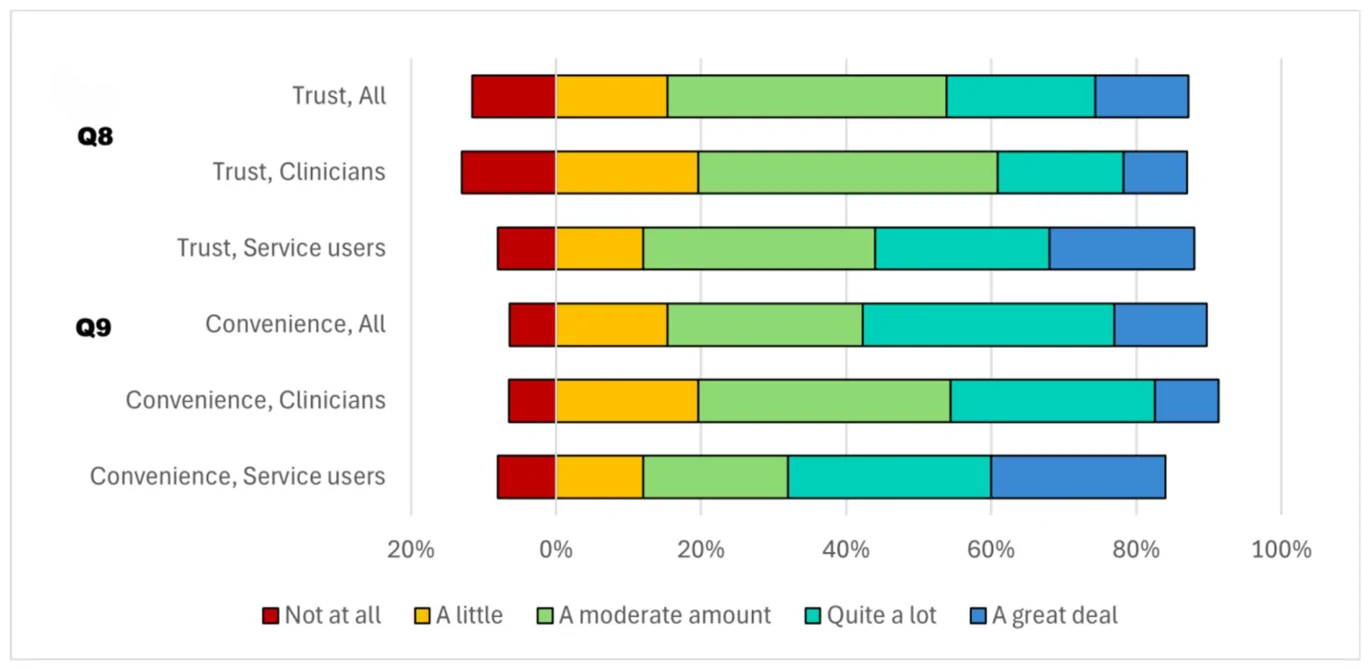
Trust (Q8) in and perceived convenience (Q9) of the AI-based OME diagnosis support system: All participants, Clinicians, and Service users.

Application of the Wilcoxon Rank Sum test and Spearman’s correlation coefficient indicated some significant differences in views between subgroups of participants (full analysis: Supplementary Materials S2, Table S2.1). In addition, a trend of more positive attitudes among service users towards the use of AI was, although not statistically significant, consistent across all evaluation measures (see Figure 2 for Q2 and Q3 and Figure 3 for Q8 and Q9). Regarding concerns, benefits, and facilitators of acceptance, the views of the two groups were statistically similar, with three notable exceptions: Clinicians were significantly more likely than service users to express concern about the flexibility of AI (C11, e.g., ‘*It is not flexible enough to be applied to every patient*’), both in general (C11_GEN: Z = −2.843, *p* = 0.004) and in the OME context (C11_OME: Z = −2.191, *p* = 0.028). In addition, concern about inaccurate predictions by AI in the OME context was significantly higher among clinicians than service users (C12_OME: Z = −2.030, *p* = 0.042). Considering that the views of clinicians and service users were statistically similar in other cases, the remaining results are reported for the full sample as a single group.

### 3.2. Survey Results on Familiarity, Approval, Trust, and Convenience Perceptions of AI in Healthcare and Ome Diagnosis

Nearly a quarter of the participants (23.2%; N = 95) reported not being familiar at all with the use of AI in healthcare. The rest indicated at least some level of familiarity (Table 2). Approval of AI was generally present: fewer than 14% of respondents expressed any level of disapproval, while over 77% reported some level of approval (Table 3). Notably, approval ratings in the OME-specific and the general contexts correlated strongly (r_s_(85) = 0.826, *p* < 0.001), suggesting consistent attitudes toward AI across different applications. Most participants (87.2%) reported at least some level of trust in the application of AI to support OME diagnosis, ranging from “a little” through most frequently moderate level (38.5%) to “a great deal” (12.8%) (Table 2). The perception of how much the AI application might improve convenience (e.g., quicker and more accurate diagnosis and treatment) was most commonly “Quite a lot” (34.6%), indicating a generally favourable view of the application’s potential benefits for patient care (Table 2).

### 3.3. Survey Results on Concerns, Perceived Benefits, and Facilitators of Acceptance-Perceptions of AI in Healthcare and Ome Diagnosis

Evaluations were conducted using Friedman tests with sign test post hoc analysis or binomial distribution for smaller data sets, with *p*-values adjusted based on the number of comparisons (Table 4). Regarding concerns about AI use (adjusted *p* = 0.001), significant differences emerged among participants both in the OME context (χ^2^(12) = 105.042, *p* < 0.001) and in the general healthcare context (χ^2^(12) = 118.836, *p* < 0.001). In the OME context, the most commonly reported concerns were *C7_Responsibility; C1_Errors; C7_Decisions; C2_Connection; C12_Inaccuracy; C10_SkillDevelopment; C13_Regulation*; and *C11_Flexibility*. In the general context they included *C7_Responsibility; C1_Errors; C12_Inaccuracy; C2_Connection; C6_Decisions; C10_SkillDevelopment; C13_Regulation;* and *C4_Dependence.* Analysis of perceived benefits (adjusted *p* = 0.002) also revealed significant differences both in the OME (χ^2^(9) = 150.050, *p* < 0.001) and the general healthcare context (χ^2^(9) = 165.119, *p* < 0.001). Most frequent perceived benefits in the OME context were *B4_Time*, *B3_Convenience*, *B3_FewerVisits*, *B2_Costs*, and *B1_DiagnosticEfficiency*. In the general healthcare context, they were *B4_Time*, *B2_Cost*, *B1_Diagnostic efficiency*, and *B3_FewerVisit.* In both contexts, communication benefits (*B8_Communication*) were expected significantly less frequently than any other option (*p* < 0.002).

Significant differences were also observed between facilitators of AI acceptance (adjusted *p* = 0.002) both in the OME (χ^2^(8) = 63.000, *p* < 0.001) and the general healthcare context (χ^2^(8) = 51.606, *p* < 0.001). In both contexts, the most frequent facilitators included accurate information (*F2_Info:Accuracy*), clear explanation of AI mechanisms (*F1_Info:Mechanism*), consideration of risks (*F4_Info:Risk consideration*), and the speed of the system (*F6_Speed*). In the OME context, information on the benefits of the system (*F3_Info:Benefits)* was commonly selected.

### 3.4. Associations Between Observed Factors

A range of significant associations were noted between the observed factors (see detailed descriptions and correlation data, including Spearman’s rho and 95%CI intervals, in Supplementary Materials S2). Higher familiarity with AI in healthcare was linked with higher approval of AI, higher concerns of regulation, and higher perceived benefits of diagnostic efficiency, unlimited resource, and data quantities. Those higher in familiarity were also less likely to see human connection as a concern, or information of experiences as a facilitator. Meanwhile, higher approval of AI was linked to higher trust in, and higher perceived convenience of, the OME diagnosis support system. Higher approval was also associated with perceiving diagnostic efficiency and accuracy as benefits of AI in healthcare. In contrast, those with lower levels of approval were more worried about human connection, rationale and justification of decisions, dependency on AI, fairness, flexibility, and clinicians’ skill development.

### 3.5. Emerging Topics from Free-Text Responses on AI in Healthcare and Ome

Some of the topics emerging in the total of 34 free-text responses overlapped with the predefined options in the evaluation questions, but new aspects were also present.

For concerns, these included confidence of doctors against AI (P39); concern about sufficient overseeing of AI (P105); errors in AI due to human errors in design (P84); loss of human control (P128); and potential inability of AI alone to increase accuracy (P42).

Further perceived benefits included accessibility of diagnostic tools, improved access to care, and improved patient outcomes (P128); consistency (P63); reduction in waiting times (P46, P119, P128); and usefulness for triage (P113). Particularly in the OME context, further perceived benefits included improved objectivity in decision-making (P93); lower invasiveness (P46); reduction in anxiety (P46); and reduction in referrals (P119).

Finally, further suggested facilitators of AI acceptance supported the main theme *Trust in technology* (see below) and related to the aspects of transparency of the AI solution (‘*designing the AI solution to be transparent so the clinician can make an informed decision to use AI or not to assist diagnosis*’ (P128)); client’s ability to opt in/out (P42); clear limits on reliance on AI (P80); and retaining human control and responsibility (P80). Particularly in the OME context, a further suggested facilitator of acceptance was further trials (P16).

In the thematic analysis, three main themes emerged: ***Trust in technology*****, *Ethics and regulation***, and ***Human abilities***.

The theme ***Trust in technology*** involved participants’ perceptions around the prospects, reliability, and risks of AI technology, suggesting hopeful but cautious predispositions regarding technological aspects such as accuracy, development, and transparency. It consisted of the following codes:

- Accuracy and errors of AI: *‘Potentially improved accuracy of diagnosis, but potentially the opposite effect too. And the same goes for the reduction of medical errors.’* (P80)*;*
- Concerns of adequate assessment and development of AI: *‘risk AI won’t continue to use data collected in later events to assess how accurate it is and improve’* (P128)*;*
- Requirement for transparent process and understood rationale of AI: *‘risk [that] decision making done by AI [is] kept in black box so clinicians don’t know why AI is making the decision … clinician must know why AI has made decision or given advice’* (P128). This code highlights the importance of transparency in AI systems, reinforcing its role as a key facilitator of AI acceptance.

The theme ***Ethics and regulation*** involved participants’ perceptions around ethical issues and regulation of the use of AI technology, suggesting a level of apprehensiveness regarding the influence of AI. It consisted of the following codes:

- Requirement for human control/overseeing: *‘Concerns about whether a clinician will have any oversight’* (P105);
- Risk of bias: ‘*risk of bias*’ (P128);
- [in OME context] Concerns of potential risks impacting children: *‘concerns about any increased risk impacting children’* (P128).

The theme ***Human abilities*** involved participants’ perceptions around the beneficial aspects, limits, and risks regarding the human aspects of using AI in healthcare such as patient interaction, confidence, and design. It consisted of the following codes:

- Human connection: ‘*I have experienced glue ear with both my children and the AI used to help diagnose it is beneficial in keeping the children involved as the clinician keeps the children involved […] and this makes it much easier. Especially for children with additional needs*’ (P10); *‘Useful as a screening tool but performing a hearing test on a young child requires skill to gain cooperation*.’ (P148);
- Confidence of doctors: ‘*Doctors will be nervous about overriding AI decision*’ (P39);
- Human error in design: ‘*Because AI tools are created by people, they have the potential to be just as fallible as a person’ (P84).*

## 4. Discussion

This pilot survey explored how clinicians and service users perceive the use of AI in healthcare, in particular in the diagnosis of OME. The investigated aspects included familiarity, approval, trust and convenience, perceived benefits, concerns, and factors that may support acceptance. The significant findings demonstrated that AI was viewed positively across the tested aspects and perceptions were generally consistent across both general healthcare and OME-specific contexts. Differences between clinicians and service users were observed in three concerns related to flexibility and accuracy of the AI-based solution: these were more common among clinicians than service users.

### 4.1. Influence of Familiarity, Experience, and Professional Role

The higher level of concern among clinicians regarding the flexibility and accuracy of AI systems aligns with the findings by Blease et al. [37], whose survey of general practitioners in the UK contradicted the optimism of many biomedical informaticians: Clinicians perceived limited potential for AI in core areas of primary care, particularly concerning communication, empathy, complex clinical reasoning, and the delivery of value-based care tasks that rely heavily on human judgment [37]. These views may reflect clinicians’ early exposure to underdeveloped AI systems and limited integration of such technologies in their practice. For example, the application of AI in primary care has lagged significantly behind secondary care, with adoption rates reported at just 23% compared to 77% by 2022 [38]. Similarly, Castagno & Khalifa [39] reported that only 36% of clinicians surveyed had encountered applications of AI tools in their clinical work. In the current study, familiarity with AI appeared relatively common, with 77% of the participants at least slightly familiar with its use in healthcare. This growing awareness in healthcare among both clinicians and service users may be linked to recent rapid advancements, which may have helped bridge the familiarity gap between clinicians and service users [40,41]. Moreover, findings in Tamori et al. [35], where clinicians were expected to have more experience than the public about AI in healthcare, indicated higher optimism about AI among clinicians. Recent research has shown that healthcare professionals tend to be more open to digital innovations [42], highlighting a nuanced relationship between experience, exposure, and attitudes, where both early familiarity and professional role shape perceptions of AI in healthcare.

### 4.2. Understanding Concerns of AI Acceptance in Healthcare

Regarding concerns, *Responsibility* and *Regulation* were frequent topics in this study, highlighting the importance of ethical discourse from this viewpoint. Similarly, concerns about inadequate AI regulation have been common in recent studies [31,33,35,39,43,44,45]. Recent literature emphasises that strong regulatory frameworks are crucial not only for patient safety and ethical use of AI, but also for building public trust and acceptance [46]. In addition, well-defined and transparent regulatory guidelines help mitigate scepticism and improve clinicians’ and patients’ confidence in AI-driven decision-making [47]. Without clear regulation, concerns about accountability, bias, and potential risks may hinder the widespread adoption and positive perception of AI technologies in clinical practice.

It is noteworthy that concerns of responsibility were not yet prominent among the previous studies, whereas in the current survey these were emphasised both in the closed questions and in the free text responses. A possible explanation could be increased consideration of the consequences of the use of AI along with the rapidly growing awareness of it. As AI continues to evolve, the integration of AI in healthcare necessitates a re-evaluation of traditional notions of accountability, as clinicians may have limited control over AI-driven decisions, challenging conventional models of moral responsibility [48]. Moreover, the ethical deployment of AI requires comprehensive education, training, and guidelines to ensure that healthcare professionals can effectively interpret and act upon AI-generated recommendations [47,49].

In the current study, *Confidentiality* and *Data leakage* were among the least common concerns, suggesting that there may be a considerable divergence of the strength of concerns among ethical aspects. The participants were less worried about data protection than about operational security (*Responsibility* and *Regulation*). The low concerns of confidentiality align with the findings in Berghea et al. [31], which suggested that participants were not widely worried about the risk of losing the confidentiality of personal data, while aspects such as possibility of errors, losing human connection, and inadequate human supervision of AI were seen as important. However, contradictory results have also been seen; for example, Castagno and Khalifa [39] highlighted worries about serious privacy issues.

*Errors*, *Inaccuracy*, and *Decisions* were emphasised concerns in the current study. These concerns are widely shared by both clinicians and patients, reflecting ongoing caution about bias and unintended consequences [19,25,26,50]. In practice, addressing these concerns depends on how clearly we communicate what the system does, how it reaches its conclusions, and how accurate it is. In our study, participants consistently identified transparency as essential, particularly in understanding how AI systems generate and support diagnostic decisions. Trust is built not simply on technical performance, but on whether systems are reliable, transparent, and clinically meaningful [19,25]. However, more information is not always better. If explanations become overly technical, they can hinder rather than support understanding [51]. What matters is communication that is clear, relevant, and aligned with the user’s level of understanding.

These findings can be interpreted through the literature on explainable AI (XAI), which distinguishes inherently interpretable models from post hoc explanations of black-box models [52,53] and recognises that explainability can support informed consent, shared decision-making, and error detection [54]. Clinicians generally seek explanations that are clinically meaningful and help them judge when to rely on, question, or override an AI recommendation [55], which is consistent with our participants’ emphasis on understanding how the AI works and why it reaches a particular result. However, explainability should complement, not replace, rigorous validation of diagnostic performance [56]. For AI-WAI applications, visualising the frequency-pressure regions contributing to classification may be useful for clinicians familiar with WAI, whereas non-specialist users and families may benefit more from a clear explanation of the result, its limitations, and the recommended next steps.

### 4.3. Perceived Benefits and the Role of Human Connection in Ai’s Acceptance in Healthcare

The most commonly perceived benefits of AI in this study were practical, focusing on time savings and financial efficiency. This aligns with previous studies suggesting significant savings in time when AI tools are applied to automate, e.g., administrative tasks [57]. Meanwhile, communication between clinician and patient was not widely considered an aspect that would benefit from AI-based solutions, despite occasional mentions to the contrary in the free-text responses. This perception might imply an expectation of a limited understanding of the AI system, which could leave people feeling vulnerable and lacking control during diagnosis. This is in line with the most common suggested facilitators, which addressed the importance of sharing more information about the AI-based systems. Other studies have identified the provision of information about AI’s functionalities as a key factor in promoting acceptance [58]. Furthermore, the suggestion was supported by the frequent reservations in the open answers regarding the extent of human oversight in the diagnostic situation. These perceptions were further reflected in the participants’ predominant concerns, which centred around operational safety, trust in technology, and the preservation of human connection.

In contrast with the concerns about trust in technology, improved diagnostic accuracy was not widely seen as a key facilitator of acceptance. In case the appreciation of diagnostic accuracy is associated with deep level knowledge about the operation of the AI-based system, this would be understandable, since only a small proportion of participants (6.3%) were very familiar with the use of AI in healthcare.

### 4.4. Limitations

This time-limited pilot study used voluntary and snowball sampling, primarily through online and professional networks. This, while effective within the restricted time, may have introduced an unknown extent of self-selection bias towards participants who were more digitally engaged or more interested in AI-related healthcare. Meanwhile, only approximately 77% of respondents reported at least some familiarity with AI, and this included participants who were only slightly familiar. Overall, self-selection may have contributed to the generally favourable approval and trust ratings observed; these should not be directly generalised to wider population before further confirmation. The modest sample size, particularly within the service-user subgroup, limited statistical power for detecting subtle between-group differences, and the findings should therefore be interpreted as preliminary and hypothesis-generating. Eligibility was based on self-reports and no additional formal exclusion criteria were applied beyond the stated eligibility requirements, which may have increased sample heterogeneity. Most clinician respondents were audiologists, with relatively limited representation from medical and primary-care practitioners, which may restrict generalisability to the broader range of potential clinical users. The service-user group comprised both carers and patients, who may differ in their experiences, risk perceptions, and decision-making roles; given the small subgroup sizes, these groups were not analysed separately but should be explored in future studies. Finally, participants evaluated a hypothetical case scenario rather than using the AI system in practice. The scenario did not explicitly specify who performed the test or who retained responsibility for the final diagnosis and management, and the trust item referred to a ‘diagnosis provided by the AI-application,’ which may have influenced participants’ interpretation of the role of AI. Although these aspects may reflect well the real-life situation particularly for service users who may not be fully aware of these details or the practical implications of them, this highlights the potentially important role of possibly complex contextual details that remain unspecified in the diagnostic situation-an issue noted also in the qualitative data. Accordingly, the findings reflect stated or prospective acceptance rather than demonstrated adoption, and future studies should evaluate the system prospectively in large and more representative samples with detailed, yet proportionate, specification of human oversight and clinical responsibility. They should also reassess acceptance after clinicians, patients, and carers have gained practical experience with the system.

## 5. Conclusions

Clinicians and service users expressed broadly favourable attitudes toward the use of AI both in OME diagnosis and in general healthcare, across aspects such as approval, trust, convenience, perceived benefits, concerns, and factors facilitating acceptance. Among the perceived key benefits were improved efficiency in terms of time and cost. However, ethical questions, particularly around responsibility and regulation, as well as technical concerns around accuracy and decision-making, were noted as common concerns. Meanwhile, most common perceived facilitators of acceptance of AI were related to available information, particularly regarding the accuracy and transparency of the AI process. To foster greater integration of AI in OME diagnosis and more widely in primary care, future implementations and promotional campaigns should involve clear consideration of the aspects highlighted by the participants. In particular, this should involve (1) Maintaining a strong principle of human oversight in AI-supported diagnosis; (2) Demonstrating this clearly to the users; (3) Specifically, developing transparent strategies to manage potential disagreements between human and AI-based suggestions; and (4) Developing and clearly explaining strategies for various potential scenarios of consequences from using AI-based suggestions in decision-making. These aspects could support future acceptance and implementation of AI-assisted OME assessment; however, its subsequent uptake and sustained use in routine clinical practice should be evaluated prospectively following real-world experience with the system.

## Figures and Tables

**Table 1:** Description of Instruments Used to Assess Participants’ Views.

Q	Instrument	Content
Q1	Familiarity	Familiarity with the use of AI applications in healthcare
Q2	Approval, general	Approval of the use of AI as a diagnostic support system in healthcare, in general
Q3	Approval, OME	Approval of the use of AI to support the diagnosis of OME
Q4	Concerns, general	Concerns regarding the use of AI in healthcare, in general
Q5	Concerns, OME	Concerns regarding the use of AI in OME diagnosis
Q6	Benefits, general	Potential benefits of using AI to support diagnoses in healthcare, in general
Q7	Benefits, OME	Potential benefits of using AI to support OME/‘Glue Ear’ diagnosis
Q8	Trust	Extent of trust in a diagnosis provided by the described AI-application, without further information
Q9	Convenience	Extent of expected improvement in OME patient experience in terms of quicker and more accurate diagnosis and treatment
Q10	Facilitators, general	Facilitators of acceptance of AI applications to support diagnosis in healthcare, in general
Q11	Facilitators, OME	Facilitators of acceptance of AI applications to support OME diagnosis

**Table 2:** Familiarity with the Use of AI in Healthcare, and Trust and Perceived Convenience Regarding the AI-Based OME Diagnostic Support System.

Aspect	Scale Level and Encoding	All	Clinicians	Service Users
**Q1 Familiarity of AI in healthcare**	Not familiar at all (1)	23.2% ± 5.5%	21.3% ± 7.6%	38.5% ± 12.2%
	Slightly familiar (2)	49.5% ± 6.6%	51.1% ± 9.3%	42.3% ± 12.4%
	Familiar (3)	21.1% ± 5.4%	21.3% ± 7.6%	15.4% ± 9.1%
	Very familiar (4)	6.3% ± 3.2%	6.4% ± 4.6%	3.8% ± 4.8%
	Median	2	2	2
	Mean	2.1 ± 0.1	2.1 ± 0.2	1.8 ± 0.2
	SD	0.831	0.824	0.834
	N	95	47	26
**Q8 Trust (OME)**	Not at all	11.5% ± 4.6%	13% ± 6.4%	8% ± 6.9%
	A little	15.4% ± 5.2%	19.6% ± 7.5%	12% ± 8.3%
	A moderate amount	38.5% ± 7.1%	41.3% ± 9.3%	32% ± 11.9%
	Quite a lot	20.5% ± 5.9%	17.4% ± 7.2%	24% ± 10.9%
	A great deal	12.8% ± 4.8%	8.7% ± 5.3%	20% ± 10.2%
	Median	3	3	3
	Mean	3.1 ± 0.2	2.9 ± 0.2	3.4 ± 0.3
	SD	1.17	1.12	1.21
	N	78	46	25
**Q9 Convenience (OME)**	Not at all	6.4% ± 3.5%	6.5% ± 4.7%	8% ± 6.9%
	A little	15.4% ± 5.2%	19.6% ± 7.5%	12% ± 8.3%
	A moderate amount	26.9% ± 6.4%	34.8% ± 9%	20% ± 10.2%
	Quite a lot	34.6% ± 6.9%	28.3% ± 8.5%	28% ± 11.5%
	A great deal	12.8% ± 4.8%	8.7% ± 5.3%	24% ± 10.9%
	Median	3	3	3
	Mean	3.2 ± 0.2	3.1 ± 0.2	3.4 ± 0.3
	SD	1.08	1.03	1.21
	N	78	46	25

Margins or error at confidence level 80%; No significant differences between clinicians and service users. N (All): Clinicians + Service users + Others.

**Table 3:** Approval of the Use of AI in General Healthcare and in OME Diagnosis.

		General Healthcare			OME		
Aspect	Scale Level and Encoding	All, General	Clinicians, General	Service Users, General	All, OME	Clinicians, OME	Service Users, OME
**Q2-Q3 Approval of the use of AI**	Strongly disapprove (1)	3.3% ± 2.4%	2.2% ± 2.8%	8.0% ± 6.9%	4.6% ± 2.9%	4.4% ± 3.9%	8.3% ± 7.2%
	Disapprove (2)	2.2% ± 2%	4.3% ± 3.8%	0% ± 0%	3.4% ± 2.5%	4.4% ± 3.9%	0% ± 0%
	Somewhat disapprove (3)	5.6% ± 3.1%	8.7% ± 5.3%	0% ± 0%	5.7% ± 3.2%	11.1% ± 6%	0% ± 0%
	Neither approve nor disapprove (4)	8.9% ± 3.8%	4.3% ± 3.8%	12.0% ± 8.3%	6.9% ± 3.5%	8.9% ± 5.4%	4.2% ± 5.2%
	Somewhat approve (5)	30.0% ± 6.2%	34.8% ± 9%	28.0% ± 11.5%	28.7% ± 6.2%	28.9% ± 8.6%	33.3% ± 12.3%
	Approve (6)	30.0% ± 6.2%	28.3% ± 8.5%	32.0% ± 11.9%	32.2% ± 6.4%	26.7% ± 8.4%	33.3% ± 12.3%
	Strongly approve (7)	17.8% ± 5.2%	17.4% ± 7.2%	16.0% ± 9.4%	18.4% ± 5.3%	15.6% ± 6.9%	20.8% ± 10.6%
	Prefer not to state	2.2% ± 2%	0% ± 0%	4.0% ± 5%	0% ± 0%	0% ± 0%	0% ± 0%
	Median	5	5	5.5	6	5	6
	Mean	5.3 ± 0.2	5.2 ± 0.3	5.2 ± 0.4	5.2 ± 0.2	5.0 ± 0.3	5.4 ± 0.4
	SD	1.43	1.45	1.59	1.54	1.61	1.58
	N	90	46	25	87	45	24

Margins of error at 80% confidence level.

**Table 4:** Concerns, Perceived Benefits, and Perceived Facilitators of Acceptance Regarding the Use of AI in Healthcare and in OME diagnosis.

		General Healthcare			OME		
Aspect	Option	All, General	Clinicians, General	Service Users, General	All, OME	Clinicians, OME	Service Users, OME
**Q4-Q5 Concerns about the use of AI**	C1-Errors	55.8% ± 6.9%	60.9% ± 9.2%	48% ± 12.8%	57% ± 7.1%	62.2% ± 9.3%	50% ± 12.6%
	C2-Connection	53.5% ± 6.9%	58.7% ± 9.3%	56% ± 12.7%	46.8% ± 7.2%	55.6% ± 9.5%	46.2% ± 12.5%
	C3-Confidentiality	17.4% ± 5.2%	15.2% ± 6.8%	16% ± 9.4%	17.7% ± 5.5%	22.2% ± 7.9%	15.4% ± 9.1%
	C4-Dependence	36% ± 6.6%	37% ± 9.1%	44% ± 12.7%	34.2% ± 6.8%	33.3% ± 9%	42.3% ± 12.4%
	C5-Fairness	22.1% ± 5.7%	26.1% ± 8.3%	20% ± 10.2%	29.1% ± 6.5%	35.6% ± 9.1%	19.2% ± 9.9%
	C6-Decisions	44.2% ± 6.9%	47.8% ± 9.4%	48% ± 12.8%	54.4% ± 7.2%	53.3% ± 9.5%	61.5% ± 12.2%
	C7-Responsibility	60.5% ± 6.7%	60.9% ± 9.2%	60% ± 12.5%	59.5% ± 7.1%	57.8% ± 9.4%	65.4% ± 11.9%
	C8-Data leakage	24.4% ± 5.9%	21.7% ± 7.8%	24% ± 10.9%	17.7% ± 5.5%	15.6% ± 6.9%	23.1% ± 10.6%
	C9-Future	16.3% ± 5.1%	13% ± 6.4%	20% ± 10.2%	15.2% ± 5.2%	13.3% ± 6.5%	19.2% ± 9.9%
	C10-Skill development	40.7% ± 6.8%	43.5% ± 9.4%	48% ± 12.8%	39.2% ± 7%	44.4% ± 9.5%	34.6% ± 11.9%
	C11-Flexibility	30.2% ± 6.3%	45.7% ± 9.4%	12% ± 8.3%	36.7% ± 6.9%	51.1% ± 9.5%	23.1% ± 10.6%
	C12-Inaccuracy	54.7% ± 6.9%	58.7% ± 9.3%	44% ± 12.7%	43% ± 7.1%	53.3% ± 9.5%	26.9% ± 11.1%
	C13-Regulation	39.5% ± 6.7%	41.3% ± 9.3%	40% ± 12.5%	39.2% ± 7%	42.2% ± 9.4%	38.5% ± 12.2%
	C14-Other	7% ± 3.5%	2.3% ± 2.8%	3.5% ± 4.7%	1.3% ± 1.6%	2.2% ± 2.8%	3.8% ± 4.8%
	N	86	46	25	79	45	26
**Q6-Q7 Perceived benefits of the use of AI**	B1-Diagnostic efficiency	70% ± 6.6%	63% ± 9.1%	76% ± 10.9%	59.5% ± 7.1%	52.2% ± 9.4%	64% ± 12.3%
	B2-Costs	71.3% ± 6.5%	76.1% ± 8.1%	64% ± 12.3%	65.8% ± 6.8%	69.6% ± 8.7%	60% ± 12.5%
	B3-Fewer visits	65% ± 6.8%	73.9% ± 8.3%	60% ± 12.5%	65.8% ± 6.8%	69.6% ± 8.7%	60% ± 12.5%
	B4-Time	86.3% ± 4.9%	89.1% ± 5.9%	84% ± 9.4%	81% ± 5.6%	84.8% ± 6.8%	80% ± 10.2%
	B5-Less errors	31.3% ± 6.6%	34.8% ± 9%	28% ± 11.5%	32.9% ± 6.8%	32.6% ± 8.8%	28% ± 11.5%
	B6-Unlimited resource	58.8% ± 7%	54.3% ± 9.4%	68% ± 11.9%	45.6% ± 7.2%	45.7% ± 9.4%	48% ± 12.8%
	B7-Data quantities	53.8% ± 7.1%	56.5% ± 9.4%	48% ± 12.8%	38% ± 7%	37% ± 9.1%	40% ± 12.5%
	B8-Communication	11.3% ± 4.5%	8.7% ± 5.3%	16% ± 9.4%	12.7% ± 4.8%	10.9% ± 5.9%	12% ± 8.3%
	B9-Convenience	57.5% ± 7.1%	60.9% ± 9.2%	52% ± 12.8%	72.2% ± 6.5%	73.9% ± 8.3%	72% ± 11.5%
	B10-Accuracy	38.8% ± 7%	37% ± 9.1%	44% ± 12.7%	39.2% ± 7%	41.3% ± 9.3%	36% ± 12.3%
	B11-Other	7.5% ± 3.8%	4.3% ± 3.8%	0% ± 0%	8.9% ± 4.1%	6.5% ± 4.7%	12% ± 8.3%
	N	80	46	25	79	46	25
**Q10-Q11 Facilitators of acceptance of AI**	F1-Info: Mechanism	76.9% ± 6.1%	82.6% ± 7.2%	76% ± 10.9%	75.6% ± 6.2%	80.4% ± 7.5%	64% ± 12.3%
	F2-Info: Accuracy	85.9% ± 5%	89.1% ± 5.9%	84% ± 9.4%	82.1% ± 5.6%	82.6% ± 7.2%	84% ± 9.4%
	F3-Info: Benefits	61.5% ± 7.1%	69.6% ± 8.7%	48% ± 12.8%	66.7% ± 6.8%	73.9% ± 8.3%	60% ± 12.5%
	F4-Info: Risk consideration	65.4% ± 6.9%	71.7% ± 8.5%	60% ± 12.5%	67.9% ± 6.8%	73.9% ± 8.3%	60% ± 12.5%
	F5-Info: Experiences	55.1% ± 7.2%	58.7% ± 9.3%	56% ± 12.7%	60.3% ± 7.1%	65.2% ± 9%	64% ± 12.3%
	F6-Speed	64.1% ± 7%	63% ± 9.1%	68% ± 11.9%	64.1% ± 7%	65.2% ± 9%	72% ± 11.5%
	F7-Lower costs	46.2% ± 7.2%	50% ± 9.4%	36% ± 12.3%	39.7% ± 7.1%	43.5% ± 9.4%	36% ± 12.3%
	F8-Diagnostic accuracy	59% ± 7.1%	65.2% ± 9%	56% ± 12.7%	56.4% ± 7.2%	56.5% ± 9.4%	64% ± 12.3%
	F9-Info: Choice	48.7% ± 7.2%	56.5% ± 9.4%	40% ± 12.5%	44.9% ± 7.2%	52.2% ± 9.4%	36% ± 12.3%
	F10-Other	5.1% ± 3.2%	0% ± 0%	12% ± 8.3%	2.6% ± 2.3%	0% ± 0%	4% ± 5%
	N	78	46	25	78	46	25

Margins of error at 80% confidence level.

## Data Availability

The data supporting the findings of this study are available from the corresponding authors upon reasonable request, subject to applicable ethical approvals and data protection requirements.

## Supplementary Materials

The following supporting information can be downloaded at https://www.mdpi.com/article/10.3390/audiolres16050157/s1, Supplementary material S1: Survey questions, scales, basis of development, factor definitions, process, and case scenario, including Table S1.1 Description and Basis of Development of Instruments and Scales; Table S1.2 Factor Definitions for Concerns and Perceived Benefits of AI, and Perceived Facilitators of Acceptance of AI; Supplementary material S2: Summary of subgroup analyses and associations between identified factors, including Table S2.1 Significant Differences in Views by Role, Gender, Age, Residence, and Ethnicity; Table S2.2 Correlations between Familiarity, Approval, Trust, Convenience, and Factors within Concerns, Benefits, and Perceived Facilitators; Table S2.3 Spearman’s rho with Confidence Intervals for Relevant Correlations.
