# Supplementary material for: Understanding AI Acceptance in Healthcare and ‘Glue Ear’ Diagnosis: A Survey of Clinicians and Service Users
Source: Audiol Res. 2026 Oct 10;16(5):157. doi: 10.3390/audiolres16050157 (PMC13701190; doi:10.3390/audiolres16050157)
Supplement: Supplementary file 1 [file audiolres-16-00157-s001.zip › audiolres-4537650-supplementary.pdf]

Supplementary material S1. Survey questions, scales, basis of development, factor definitions, process, and case scenario

Question scales and basis of development

Table S1.1

Description and Basis of Development of Instruments and Scales

Q	Instrument	Content	Scale	Options	Basis	Original item
Q1	Familiarity	Familiarity with the use of AI applications in healthcare	4-point Likert + PNTS	Not familiar at all / Slightly familiar / Familiar / Very familiar / Prefer not to state	e	*Knowledge of AI in medicine*
Q2	Approval, general*	Approval of the use of AI as a diagnostic support system in healthcare, in general	7-point Likert + PNTS	Strongly disapprove ... Strongly approve / PNTS	a	Q9
Q3	Approval, OME	Approval of the use of AI to support the diagnosis of OME	7-point Likert + PNTS	Strongly disapprove ... Strongly approve / PNTS	a	Q9
Q4	Concerns, general	Concerns regarding the use of AI in healthcare, in general	Multiple choice incl Other	C1-C14**	a / c / e	Q15, Q21 / PRA1, PR2, PPC4, PPC5, PCB1, PCB3, PCB4, PUS2, PL3 / *Concern about data leakage* / Q10
Q5	Concerns, OME	Concerns regarding the use of AI in OME diagnosis	Multiple choice incl Other	C1-C14**	a / c / d / e	Q15, Q21 / PRA1, PR2, PPC4, PPC5, PCB1, PCB3, PCB4, PUS2, PL3 / *Concern about data leakage* / Q10
Q6	Benefits, general	Potential benefits of using AI to support diagnoses in healthcare, in general	Multiple choice incl Other	B1-B11**	b / d / e	NMPE1 / Q6 / Efficiency
Q7	Benefits, OME	Potential benefits of using AI to support OME / 'Glue Ear' diagnosis	Multiple choice incl Other	B1-B11**	b / d / e	NMPE1 / Q6 / *Efficiency*
Q8	Trust	Extent of trust in a diagnosis provided by the described AI-application, without further information	5-point Likert + PNTS	Not at all / A little / A moderate amount / Quite a lot / A great deal / Prefer not to state	f	n/a

Q9	Convenience	Extent of expected improvement in OME patient experience in terms of quicker and more accurate diagnosis and treatment	5-point Likert + PNTS	Not at all / A little / A moderate amount / Quite a lot / A great deal / Prefer not to state	e	*Efficiency*
Q10	Facilitators, general	Facilitators of acceptance of AI applications to support diagnosis in healthcare, in general	Multiple choice incl. Other	F1-F10**	a	Q14
Q11	Facilitators, OME	Facilitators of acceptance of AI applications to support OME diagnosis	Multiple choice incl. Other	F1-F10**	a	Q14

OME: Otitis media with effusion. PNTS: Prefer not to state. a: Berghea et al., 2024 [31]; b: Cornelissen et al., 2022 [32]; c: Esmaeilzadeh, 2020 [33]; d: Oh et al., 2019 [34]; e: Tamori et al., 2022 [35]; f: Research team, PPIE group, and pilot test group.
* Response obligatory for proceeding in the survey; in other questions, response was requested but not obligatory. ** Table S1.2

Assessment questions, wording

Q1 How familiar are you with the use of Artificial Intelligence (AI) applications in healthcare?
Please answer the following questions based on the case scenario presented, your views of artificial intelligence, and your experiences of OME / 'Glue Ear'.

Q2 To what extent do you approve of the use of AI as a diagnostic support system **in healthcare, in general**?

Q3 To what extent do you approve of the use of AI to support the diagnosis of **OME / 'Glue Ear'**?

Q4 What are your main concerns regarding the use of AI **in healthcare, in general**? (Select as many as you wish)

Q5 What are your main concerns regarding the use of AI in **OME / 'Glue Ear'** diagnosis? (Select as many as you wish)

Q6 What benefits might there be of using AI to support diagnoses **in healthcare, in general**? (Select as many as you wish)

Q7 What benefits might there be of using AI to support **OME / 'Glue Ear' diagnosis**? (Select as many as you wish)

Q8 To what extent would you trust a diagnosis provided by the AI-application (the AI-based diagnostic system for OME / 'Glue Ear' diagnosis, described in the case scenario and illustrated in the image earlier in this survey), without further information?

Q9 How much do you feel that using the described AI application to help diagnose OME / 'Glue Ear' could improve the patient experience (in terms of quicker and more accurate diagnosis and treatment)?

Q10 What do you think might increase acceptance of AI applications to support diagnosis **in healthcare, in general**? (Select as many as you wish)

Q11 What do you think might increase acceptance of AI applications to support **OME / 'Glue Ear' diagnosis**? (Select as many as you wish)

Factor definitions

Table S1.2

Factor Definitions for Concerns and Perceived Benefits of AI, and Perceived Facilitators of Acceptance of AI

Concerns

C1 - Errors	AI can make errors
C2 - HumanConnection	Using AI loses the human connection between the patient and the doctor
C3 - Confidentiality	The use of AI will lead to the loss of confidentiality of personal data
C4 - Dependence	The use of AI will create excessive dependence on technology
C5 - Fairness	With AI, one can no longer be sure that medical decisions are fair and the same for everyone
C6 - Decisions	With AI, it becomes more difficult to understand who makes the decisions and why a certain decision was made
C7 - Responsibility	It is not clear who becomes responsible if AI tools offer wrong recommendations
C8 - DataLeakage	Data leakage
C9 - Future	Uncertainty about the future development of AI
C10 - SkillDevelopment	Clinicians' diagnostic skills will suffer
C11 - Flexibility	It is not flexible enough to be applied to every patient
C12 - Inaccuracy	Inaccurate predictions by AI
C13 - Regulation	I am concerned that the safety and efficacy of AI medical tools are not regulated clearly
C14 - Other	Other

Benefits

B1 - DiagnosticEfficiency	Doctors could accomplish diagnostic tasks more efficiently
B2 - Costs	Reduced healthcare costs
B3 - FewerVisits	Reduction in the number of required appointments
B4 - Time	Could save time
B5 - Less errors	AI can help reduce medical errors
B6 - UnlimitedResource	AI has no emotional exhaustion nor physical limitation
B7 - DataQuantities	AI can deliver vast amounts of clinically relevant high-quality data in real time
B8 - Communication	Improved communication between clinician and client
B9 - Convenience	Convenience
B10 - Accuracy	Improved accuracy of diagnosis
B11 - Other	Other

Facilitators

F1 - Info: Mechanism	More information on how the AI application works
F2 - Info: Accuracy	More information on the accuracy of the AI application
F3 - Info: Benefits	More information on the benefits of the use of the AI application
F4 - Info: RiskConsideration	More information on how the risks of the use of the AI application are taken into account
F5 - Info: Experiences	Hearing about other users' experiences
F6 - Speed	Shorter waiting times and faster appointments
F7 - LowerCosts	Reduced costs for patient
F8 - DiagnosticAccuracy	Higher accuracy in diagnosis
F9 - Info: Choice	Informing clients that they can choose whether to use AI
F10 - Other	Other

Process

Participants started the survey by following an electronic link, after which they were asked for their role regarding OME and presented with the respective participant information sheet of the study: Based on the suggestions of the PPIE panel, the participant information section was separated into two different versions – one for clinicians and one for service users, with slightly different content considering the different contexts of these respondents. Both versions were carefully worded to enhance clarity, based on the PPIE group feedback and led by the lead of the PPIE group. After the information sheet, the participants were asked to read and tick the check boxes of an electronic consent form, also carefully worded for clarity based on the feedback. This confirmed the participants' informed consent to participate and was required before the participants could proceed to the study. Participants were then presented the withdrawal code and asked to note this down, while it was also noted that the code would be presented again at the end of the survey. After this, clinicians were asked their profession. All participants were then asked about their familiarity with the use of AI applications in healthcare, presented with the OME diagnosis case scenario, and asked the evaluation questions. The survey ended with the collection of the demographic data and the repeated code of withdrawal, and with a final question offering the participants the opportunity to freely comment on anything that was on their mind before submitting the survey. The duration of the survey was approximately 10 minutes.

Case scenario

Please read the following description of the diagnosis of OME / 'Glue Ear', using the support of an AI-based diagnostic system.

****

[Case scenario] Dr. Smith, a community paediatrician, is seeing a 5-year-old girl named Emily. Emily's mother expresses concerns about her frequent ear infections and hearing difficulties, such as struggles hearing speech, the television, and in school. Dr. Smith decides to investigate further for possible otitis media with effusion (OME, also known as 'Glue Ear', a common condition in children where fluid accumulates in the middle ear). Dr. Smith assesses Emily's symptoms and medical history, followed by a simple, quick, and non-invasive hearing test. The results are interpreted by an AI-based diagnostic system, suggesting a 85% likelihood of 'Glue Ear' as the underlying condition.*

****

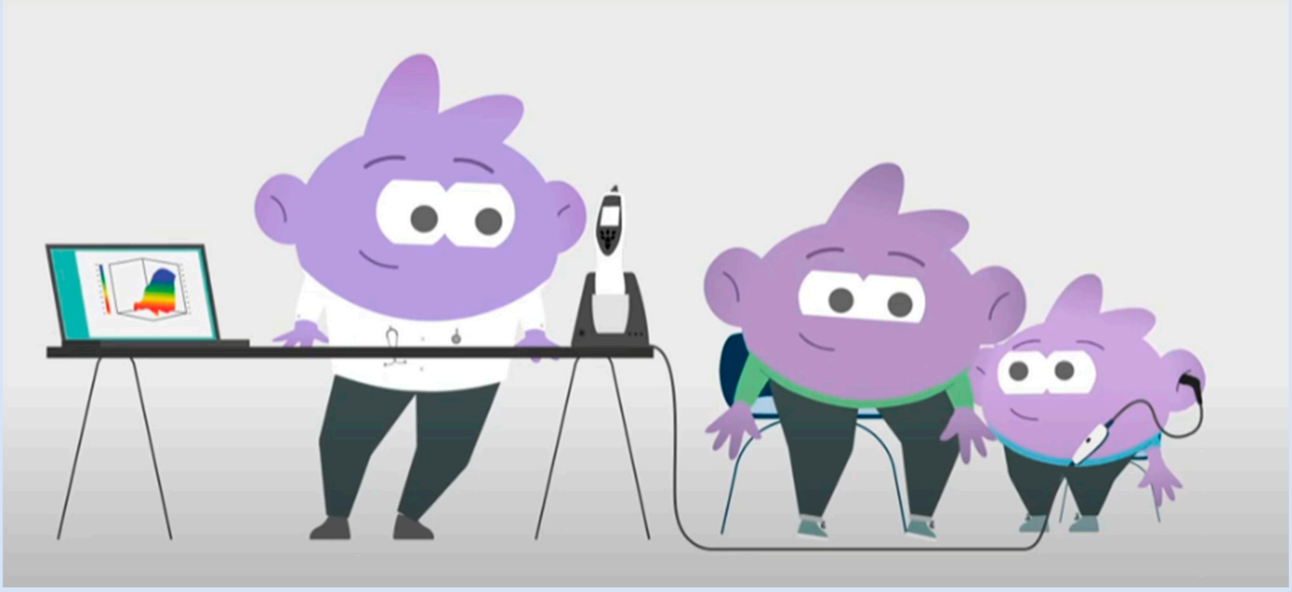

** Note: The AI-based diagnostic system that is used is a combination of a simple hearing test device and a user-friendly graphical software. The software automatically provides a diagnostic suggestion based on an ear function test, as illustrated in the image above. For this test, a small probe is placed in the patient's ear canal. The probe sends quiet 'click' sounds into the ear and records the responses from the ear canal. The test is non-invasive and very quick.*

Supplementary material S2. Summary of subgroup analyses and associations between identified factors

Table S2.1 shows significant differences between subgroups for all assessed factors. Table S2.2 shows significant associations between the applicable assessments and familiarity, approval, trust, and confidence, and Table S2.3 shows the related confidence intervals. (Differences between subgroups: Wilcoxon Rank Sum test; Correlation of factors with age: Spearman's correlation; Effect size estimates: Spearman's rho).

Table S2.1

Significant Differences in Views by Role, Gender, Age, Residence, and Ethnicity

Q	Scale / Option	Role	Gender	Age	Residence	Ethnicity
**Q1 Familiarity**	Scale 1-4				●	
**Q2 Approval / General**	Scale 1-7		●●			
**Q3 Approval / OME**	Scale 1-7		●●		●	
**Q4 Concerns / General**	CG1 - Errors					
	CG2 - Connection					
	CG3 - Confidentiality					
	CG4 - Dependence					
	CG5 - Fairness					
	CG6 - Decisions			●		
	CG7 - Responsibility					
	CG8 - Data leakage					
	CG9 - Future					
	CG10 - Skill development					
	CG11 - Flexibility	●●				
	CG12 - Inacuracy					
	CG13 - Regulation		●			
	CG14 - Other					
**Q5 Concerns / OME**	CO1 - Errors					
	CO2 - Connection					
	CO3 - Confidentiality			●		●
	CO4 - Dependence					
	CO5 - Fairness			●		
	CO6 - Decisions					
	CO7 - Responsibility					●
	CO8 - Data leakage					
	CO9 - Future					
	CO10 - Skill development					
	CO11 - Flexibility	●	●			
	CO12 - Inaccuracy	●				
	CO13 - Regulation		●●			
	CO14 - Other					

**Q6 Perceived benefits / General**	BG1 - Diagnostic efficiency					
	BG2 - Costs					
	BG3 - Fewer visits					
	BG4 - Time					
	BG5 - Less errors		●			
	BG6 - Unlimited resource					
	BG7 - Data quantities					
	BG8 - Communication					
	BG9 - Convenience					
	BG10 - Accuracy					
	BG11 - Other					
**Q7 Perceived benefits / OME**	BO1 - Diagnostic efficiency				●●	
	BO2 - Costs					
	BO3 - Fewer visits					
	BO4 - Time					
	BO5 - Less errors		●			
	BO6 - Unlimited resource					
	BO7 - Data quantities					
	BO8 - Communication					
	BO9 - Convenience					
	BO10 - Accuracy					●
	BO11 - Other					
**Q8 Trust**	Scale 1-5					
**Q9 Convenience**	Scale 1-5					
**Q10 Facilitators of acceptance / General**	FG1 - Info: Mechanism					
	FG2 - Info: Accuracy					
	FG3 - Info: Benefits					
	FG4 - Info: Risk consideration					
	FG5 - Info: Experiences					
	FG6 - Speed					
	FG7 - Lower costs					●●
	FG8 - Diagnostic accuracy					
	FG9 - Info: Choice					
	FG10 - Other					
**Q11 Facilitators of acceptance / OME**	FO1 - Info: Mechanism					
	FO2 - Info: Accuracy					
	FO3 - Info: Benefits					
	FO4 - Info: Risk consideration		●			
	FO5 - Info: Experiences					
	FO6 - Speed					
	FO7 - Lower costs					●●
	FO8 - Diagnostic accuracy					
	FO9 - Info: Choice					
	FO10 - Other					

●: small effect size (0.1 ≤ rho < 0.3); ●●: medium effect size (0.3 ≤ rho < 0.5)

Table S2.2

Correlations between Familiarity, Approval, Trust, Convenience, and Factors within Concerns, Benefits, and Perceived Facilitators

Q	Scale / Option	Q1 Familiarity	Q2 Approval / General	Q3 Approval / OME	Q8 Trust	Q9 Convenience
**Q1 Familiarity**	Scale 1-4		↑	↑		
**Q2 Approval / General**	Scale 1-7	↑		↑	↑	↑
**Q3 Approval / OME**	Scale 1-7	↑	↑		↑	↑
**Q4 Concerns / General ***	CG1 - Errors					
	CG2 - HumanConnection		↓		↓	↓
	CG3 - Confidentiality					
	CG4 - Dependence					
	CG5 - Fairness		↓			
	CG6 - Decisions			↓		
	CG7 - Responsibility					
	CG8 - DataLeakage					
	CG9 - Future					
	CG10 - SkillDevelopment					
	CG11 - Flexibility					
	CG12 - Inaccuracy				↓	
	CG13 - Regulation	↑				
	CG14 - Other					
**Q5 Concerns / OME ***	CO1 - Errors					
	CO2 - HumanConnection	↓	↓	↓		
	CO3 - Confidentiality		↓			
	CO4 - Dependence		↓	↓		
	CO5 - Fairness			↓		
	CO6 - Decisions		↓	↓		↓
	CO7 - Responsibility					
	CO8 - DataLeakage					
	CO9 - Future					
	CO10 - SkillDevelopment		↓	↓	↓	↓
	CO11 - Flexibility		↓	↓		↓
	CO12 - Inaccuracy				↓	↓
	CO13 - Regulation					
	CO14 - Other					
**Q6 Perceived benefits / General ***	BG1 - DiagnosticEfficiency	↑	↑	↑	↑	↑
	BG2 - Costs					
	BG3 - FewerVisits					
	BG4 - Time					
	BG5 - Less errors					↑
	BG6 - UnlimitedResource					
	BG7 - DataQuantities		↑	↑		

	BG8 - Communication					
	BG9 - Convenience					
	BG10 - Accuracy		↑	↑		
	BG11 - Other					
**Q7 Perceived benefits / OME ***	BO1 - DiagnosticEfficiency		↑	↑	↑	↑
	BO2 - Costs					
	BO3 - FewerVisits					
	BO4 - Time					
	BO5 - Less errors		↑			
	BO6 - UnlimitedResource	↑				
	BO7 - DataQuantities	↑				
	BO8 - Communication					
	BO9 - Convenience					
	BO10 - Accuracy		↑	↑		
	BO11 - Other					
**Q8 Trust**	Scale 1-5		↑	↑		↑
**Q9 Convenience**	Scale 1-5		↑	↑	↑	
**Q10 Facilitators of acceptance / General ***	FG1 - Info: Mechanism					
	FG2 - Info: Accuracy					
	FG3 - Info: Benefits					
	FG4 - Info: RiskConsideration					
	FG5 - Info: Experiences					
	FG6 - Speed					
	FG7 - LowerCosts					
	FG8 - DiagnosticAccuracy					
	FG9 - Info: Choice					
	FG10 - Other					
**Q11 Facilitators of acceptance / OME ***	FO1 - Info: Mechanism					
	FO2 - Info: Accuracy					
	FO3 - Info: Benefits					
	FO4 - Info: RiskConsideration					
	FO5 - Info: Experiences	↓				
	FO6 - Speed				↑	
	FO7 - LowerCosts					
	FO8 - DiagnosticAccuracy					
	FO9 - Info: Choice					
	FO10 - Other					
Total significant correlations		8	18	16	10	11

* Likelihood of selection

↑ Positive correlation; ↓ Negative correlation

White:	Small effect size (0.1 ≤ rho < 0.3)
Light green:	Moderate effect size (0.3 ≤ rho < 0.5)
**Dark green:**	Large effect size (rho > 0.5)

Table S2.3

Spearman's rho with Confidence Intervals for Relevant Correlations

Confidence Intervals of Spearman's rho

	Spearman's rho	Significance(2-tailed)	95% Confidence Intervals (2-tailed)[a,b]	
			Lower	Upper
Age - Role	0.296	0.012	0.060	0.500
Age - CG6_Decisions	-0.248	0.029	-0.451	-0.020
Age - CO3_Confidentiality	-0.251	0.028	-0.455	-0.022
Age - CO5_Fairness	-0.245	0.032	-0.450	-0.016
Gender - Approval_Gen	0.384	0.001	0.166	0.567
Gender - Approval_OME	0.358	0.002	0.135	0.547
Gender - CG13_Regulation	0.284	0.013	0.055	0.484
Gender - CO11_Flexibility	-0.231	0.046	-0.441	0.002
Gender - CO13_Regulation	0.326	0.004	0.100	0.520
Gender - BG5_LessErrors	0.233	0.042	0.002	0.441
Gender - BO5_LessErrors	0.277	0.016	0.048	0.478
Gender - FO4_InfoRiskConsideration	-0.277	0.016	-0.478	-0.048
Residence - Role	-0.492	0.000	-0.663	-0.274
Residence - Familiarity	0.273	0.023	0.031	0.484
Residence - Approval_OME	-0.281	0.021	-0.494	-0.036
Residence - BO1_DiagnosticEfficiency	-0.307	0.010	-0.512	-0.069
Role - CG11_Flexibility	-0.340	0.004	-0.536	-0.109
Role - CO11_Flexibility	-0.264	0.027	-0.475	-0.024
Role - CO12_Inaccuracy	-0.244	0.041	-0.459	-0.003
Familiarity - Approval_Gen	0.285	0.007	0.074	0.472
Familiarity - Approval_OME	0.252	0.019	0.037	0.444
Familiarity - CG13_Regulation	0.222	0.040	0.004	0.420
Familiarity - CO2_Connection	-0.246	0.029	-0.448	-0.019
Familiarity - BG1_DiagnosticEfficiency	0.241	0.031	0.016	0.443
Familiarity - BO6_UnlimitedResource	0.244	0.030	0.017	0.446
Familiarity - BO7_DataQuantities	0.249	0.027	0.023	0.451
Familiarity - FO5_InfoExperiences	-0.251	0.026	-0.454	-0.024
Approval_Gen - Approval_OME	0.826	0.000	0.742	0.884
Approval_Gen - Trust_OME	0.413	0.000	0.202	0.587
Approval_Gen - Convenience_OME	0.440	0.000	0.222	0.616
Approval_Gen - CG2_Connection	-0.286	0.008	-0.475	-0.071
Approval_Gen - CG5_Fairness	-0.291	0.007	-0.480	-0.077
Approval_Gen - CO2_Connection	-0.402	0.000	-0.578	-0.191
Approval_Gen - CO3_Confidentiality	-0.252	0.026	-0.455	-0.025
Approval_Gen - CO4_Dependence	-0.232	0.041	-0.438	-0.003
Approval_Gen - CO6_Decisions	-0.252	0.026	-0.455	-0.025
Approval_Gen - CO10_SkillDevelopment	-0.352	0.002	-0.537	-0.134
Approval_Gen - CO11_Flexibility	-0.320	0.004	-0.511	-0.098

Approval_Gen - BG1_DiagnosticEfficiency	0.389	0.000	0.178	0.567
Approval_Gen - BG7_DataQuantities	0.426	0.000	0.220	0.596
Approval_Gen - BG10_Accuracy	0.375	0.001	0.161	0.555
Approval_Gen - BO1_DiagnosticEfficiency	0.403	0.000	0.192	0.578
Approval_Gen - BO5_LessErrors	0.306	0.007	0.082	0.499
Approval_Gen - BO10_Accuracy	0.353	0.002	0.135	0.538
Approval_OME - Trust_OME	0.547	0.000	0.361	0.691
Approval_OME - Convenience_OME	0.552	0.000	0.359	0.701
Approval_OME - CO2_Connection	-0.319	0.005	-0.511	-0.095
Approval_OME - CO4_Dependence	-0.267	0.019	-0.469	-0.039
Approval_OME - CO5_Fairness	-0.236	0.039	-0.443	-0.006
Approval_OME - CO6_Decisions	-0.321	0.004	-0.513	-0.098
Approval_OME - CO10_SkillDevelopment	-0.275	0.015	-0.475	-0.048
Approval_OME - CO11_Flexibility	-0.362	0.001	-0.547	-0.144
